# Bufotalin enhances apoptosis and TMZ chemosensitivity of glioblastoma cells by promoting mitochondrial dysfunction via AKT signaling pathway

**DOI:** 10.18632/aging.205883

**Published:** 2024-05-28

**Authors:** Zhansheng Zhu, Shanwen Liang, Yu Hong, Yangzhi Qi, Qian Sun, Xinyi Zhu, Yuxin Wei, Yang Xu, Qianxue Chen

**Affiliations:** 1 Department of Neurosurgery, Renmin Hospital of Wuhan University, Wuhan University, Wuhan, Hubei 430060, China; 2 Central Laboratory, Renmin Hospital of Wuhan University, Wuhan, Hubei 430060, China

**Keywords:** bufotalin, mitochondrial dysfunction, ROS, AKT, apoptosis, TMZ sensitivity

## Abstract

Glioblastoma multiforme (GBM) is the most prevalent and lethal primary intracranial neoplasm in the adult population, with treatments of limited efficacy. Recently, bufotalin has been shown to have anti-cancer activity in a variety of cancers. This investigation aims to investigate the effect of bufotalin on GBM and elucidate its potential underlying mechanism. Our results show that bufotalin not only inhibits the proliferation and epithelial-mesenchymal transition (EMT) but also triggers apoptosis in GBM cells. The result of RNA-seq indicated that bufotalin could induce mitochondrial dysfunction. Moreover, our observations indicate that bufotalin induces an excessive accumulation of intracellular reactive oxygen species (ROS) in GBM cells, leading to mitochondrial dysfunction and the dephosphorylation of AKT. Moreover, bufotalin improved TMZ sensitivity of GBM cells *in vitro* and *in vivo*. In conclusion, bufotalin enhances apoptosis and TMZ chemosensitivity of glioblastoma cells by promoting mitochondrial dysfunction via AKT signaling pathway.

## INTRODUCTION

Glioblastoma is the most common and lethal primary intracranial neoplasm in adults, accounting for approximately fifty percent of all malignant intracranial tumors. Despite the implementation of conventional therapeutic modalities such as tumor resection, adjuvant radiotherapy, and cyclic temozolomide chemotherapy in recent times, patients exhibit a survival rate of only 40.6% survival within the initial year, with the five-year survival rate dropping to a minimum of 5.6% [1, 2]. Dysregulated control of cell death is considered to be a critical factor contributing to the resistance of glioblastoma to temozolomide [3, 4]. Therefore, fostering glioblastoma cell death and enhancing its sensitivity to temozolomide hold substantial importance in the treatment of glioblastoma.

Apoptosis is a type of programmed cell death characterised by nuclear condensation and the formation of apoptotic bodies, which is followed by phagocytosis [3]. Mitochondria are major centres of cellular energy metabolism and also crucial organelles in the regulation of cancer cell apoptosis [5]. Under various apoptotic signals such as DNA damage [6] and ROS accumulation [7], mitochondria of glioblastoma cells undergo significant structural and functional changes, resulting in the release of various apoptotic mediators, such as cytochrome C and apoptosis-inducing factor, into the cytoplasm, initiating the apoptotic programme through the activation of caspases or other apoptosis-related proteins [8]. Thus, inducing mitochondrial dysfunction in GBM cells may be a potential strategy for treating GBM and reducing TMZ resistance. For instance, Zheng et al. found that SW33 triggered apoptosis dependent on mitochondrial pathways and initiated cellular autophagy via the PI3K/AKT/mTOR and AMPK/mTOR signaling cascades [9]. The B cell lymphoma 2 (Bcl2) protein family assumes a pivotal role as a “switch” in governing mitochondrial apoptosis regulation, and it regulates the stability of mitochondrial structure and function through synergistic interaction with other apoptotic proteins [10]. Liang et al. have shown that PKM2-dependent phosphorylation and stabilisation of Bcl2 plays a critical role in the resistance of glioma cells to oxidative stress-induced apoptosis [11].

In recent years, natural products have garnered increasing attention in the field of cancer treatment owing to their myriads of biological activities and advantageous health benefits [12]. ChanSu, commonly known as toad venom or Venenum Bufonis, represents a valuable traditional Chinese medicine derived from the desiccated secretions of the Asiatic toad or black-spectacled toad belonging to the Bufonidae family. Among the constituents of ChanSu, bufadienolides and indole alkaloids (bufotenines) are considered to be two important bioactive substances [13]. Recently, more and more studies have demonstrated that the effects of bufadienolides are associated with various mechanisms. For instance, the cytotoxic impact of bufalin was linked to an elevated G2/M cycle arrest and diminished cell proliferation in U87MG and U251 cells. Additionally, bufalin disrupted the mitochondrial membrane potential, resulting in decreased oxygen consumption and ATP production [14]. Lv et al. have shown that arenobufagin effectively induces apoptosis in ESCC primarily by initiating caspase activation through both intrinsic and extrinsic pathways, mediated via the p53 pathway [15]. Recently, the anticancer activity of bufotalin has been found in various cancers. Bufotalin demonstrates the capacity to induce cell cycle disruption at the G2/M juncture and initiate cellular apoptosis. This results in the inhibition of proliferative activity in A375 cells, suggesting that bufotalin holds promise as a potential candidate for the treatment of melanoma [16]. By accelerating the degradation of GPX4 and elevating the intracellular concentration of Fe2+, bufotalin possesses the capacity to initiate lipid peroxidation and induce ferroptosis. This makes bufotalin a promising candidate for the development of anti-tumor agents that specifically target ferroptosis [17]. However, the effect and the underlying molecular mechanism in GBM cells remain largely elusive.

Here, we found that bufotalin can significantly inhibit EMT and promote apoptosis of GBM cells. The potential mechanism is that bufotalin induces mitochondrial dysfunction and inhibits the phosphorylation of AKT by increasing the production of ROS. Furthermore, we have shown that bufotalin can also increase the chemosensitivity of GBM cells to TMZ *in vivo* and *in vitro*.

## RESULTS

### Bufotalin inhibits cell proliferation in GBM cells

**Figure 1:**
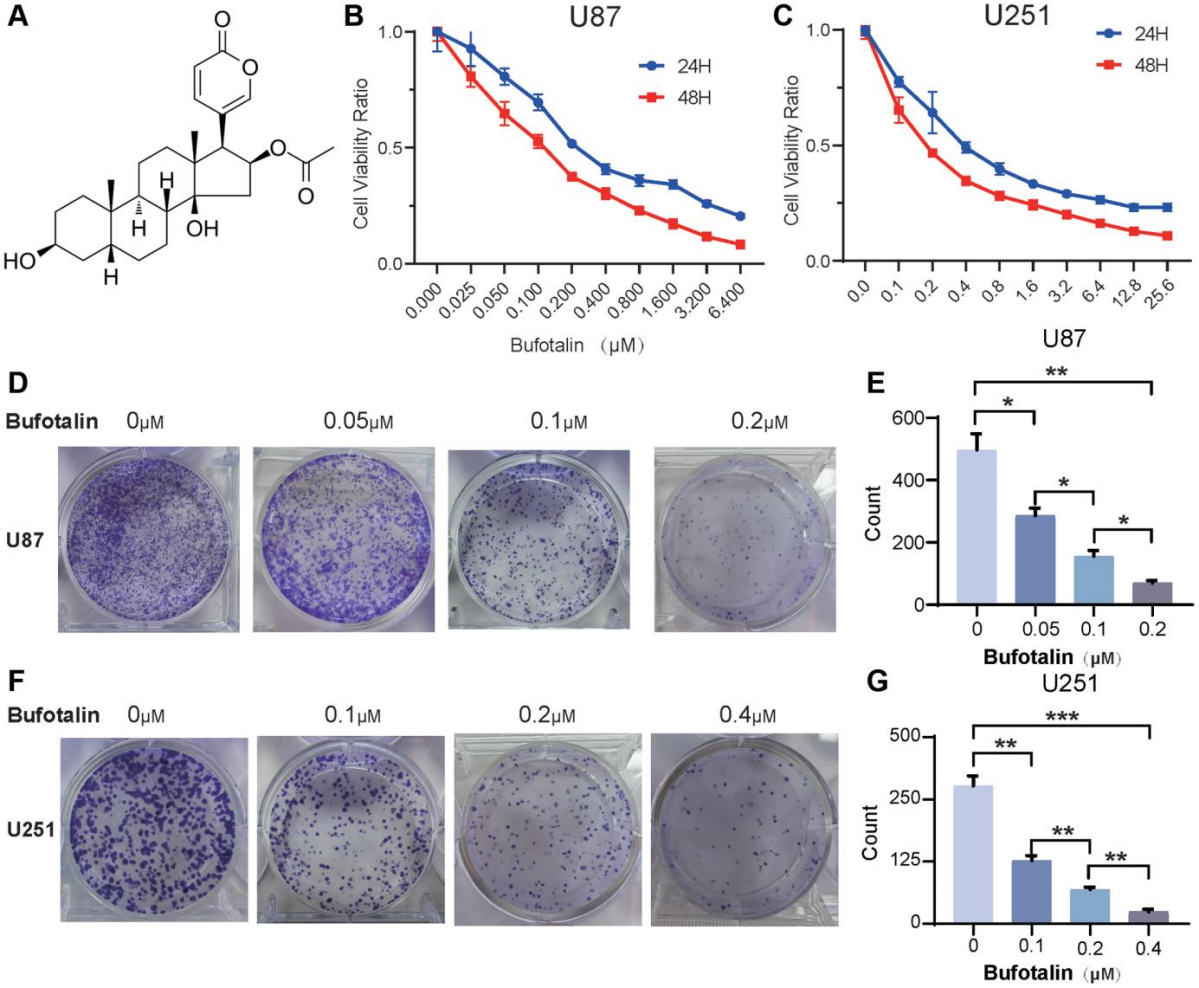
**Bufotalin inhibits cell proliferation in GBM cells.** (**A**) The molecular structure of bufotalin. (**B**, **C**) CCK-8 assay was used to determine the cell viability of U87 and U251 cells treated with bufotalin for 24 h and 48 h. (**D**–**G**) Bufotalin suppressed U87 and U251 cells’ colony formation. The data are representative of three independent experiments and are presented as the mean ± SD. Significant differences compared with the control are indicated by ^*^p < 0.05, ^**^p < 0.01, and ^***^p < 0.001.

The molecular structure of bufotalin was shown in Figure 1A. U87 and U251 cells were treated with different concentrations of bufotalin. According to CCK8, we found that bufotalin significantly inhibited the viability of U87 and U251 cells in a dose and time-dependent manner with 113.2 nM (U87) and 199.5 nM (U251) at 24 h and with 102.3 nM (U87) and 134 nM (U251) at 48 h (Figure 1B, 1C). The colony formation assay was performed and the results showed that bufotalin had a significant dose-dependent effect on reducing the number of colonies formed by U87 and U251 cells (Figure 1D–1G).

### Functional enrichment analysis of DEGs after treatment of bufotalin according to RNA-seq

**Figure 2:**
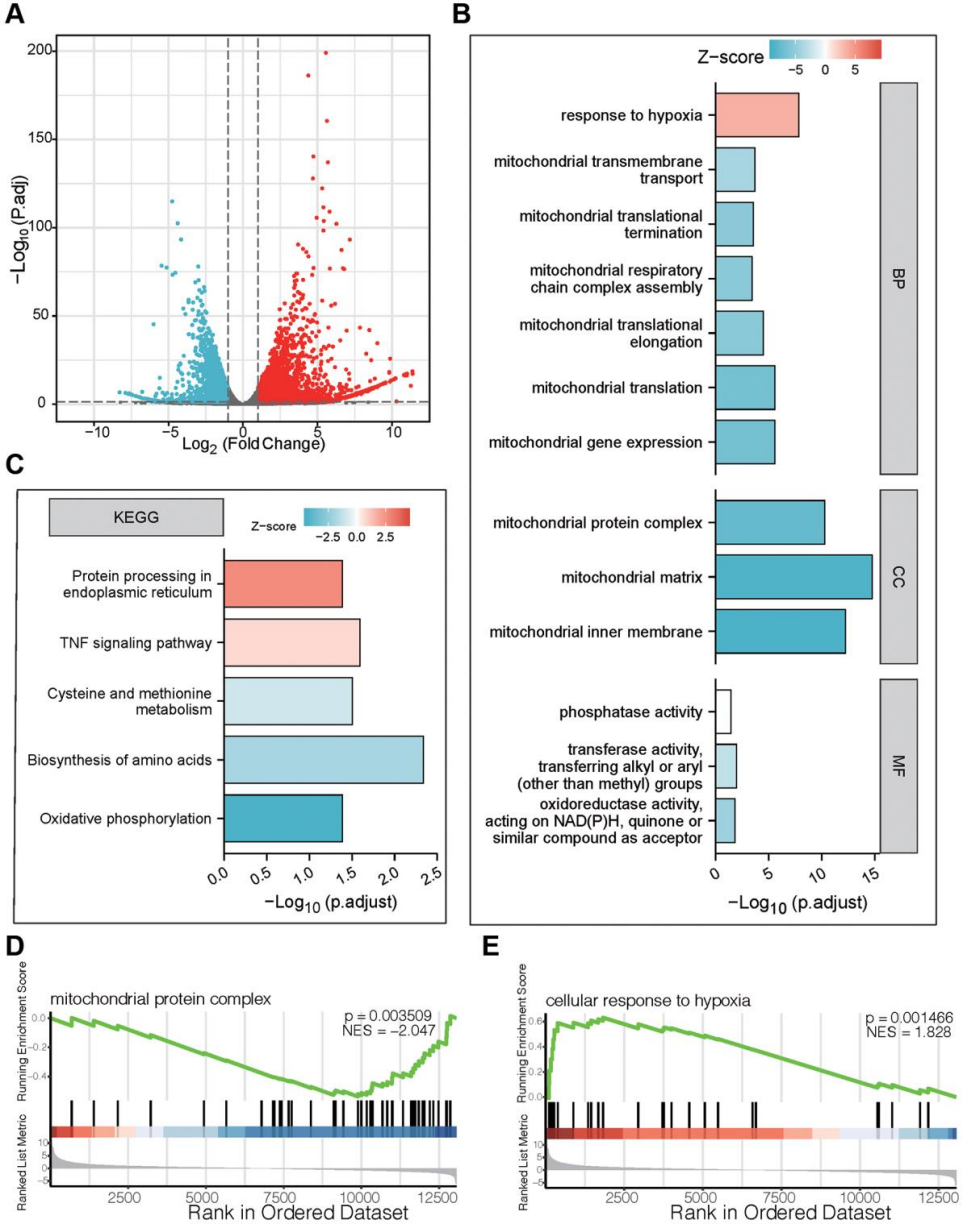
**Functional enrichment analysis of DEGs after treatment of bufotalin according to RNA-seq in U87 cells.** (**A**) volcano plot. (**B**) GO enrichment analysis of downregulated DEGs. (**C**) KEGG enrichment analysis of upregulated DEGs. (**D**, **E**) GSEA enrichment analysis between the control group and the bufotalin group.

In order to further explore the potential mechanism of bufotalin on GBM cells, the U87 cells were treated with DMSO as a control group and bufotalin for 24 h, and then transcriptomic sequencing was conducted. First, we performed differentially expressed gene analysis and generated a volcano plot (Figure 2A). And the number of upregulated genes was 2942, while the number of downregulated genes was 2353 in bufotalin group compared with control group. Next, we performed GO analysis and the results showed that the mitochondrial-related signaling pathway was significantly downregulated in the bufotalin group, suggesting its potential disruption of mitochondrial function (Figure 2B). Furthermore, we conducted KEGG analysis and found that amino acid synthesis and oxidative phosphorylation were downregulated in the bufotalin group (Figure 2C). Besides, upregulated and downregulated genes were used to perform GSEA and the results showed that mitochondrial protein complex was downregulated and cellular response to hypoxia was upregulated (Figure 2D, 2E). Overall, the results of RNA-seq demonstrated that bufotalin could lead to the dysfunction of mitochondria.

### Bufotalin attenuated the EMT process of GBM cells

**Figure 3:**
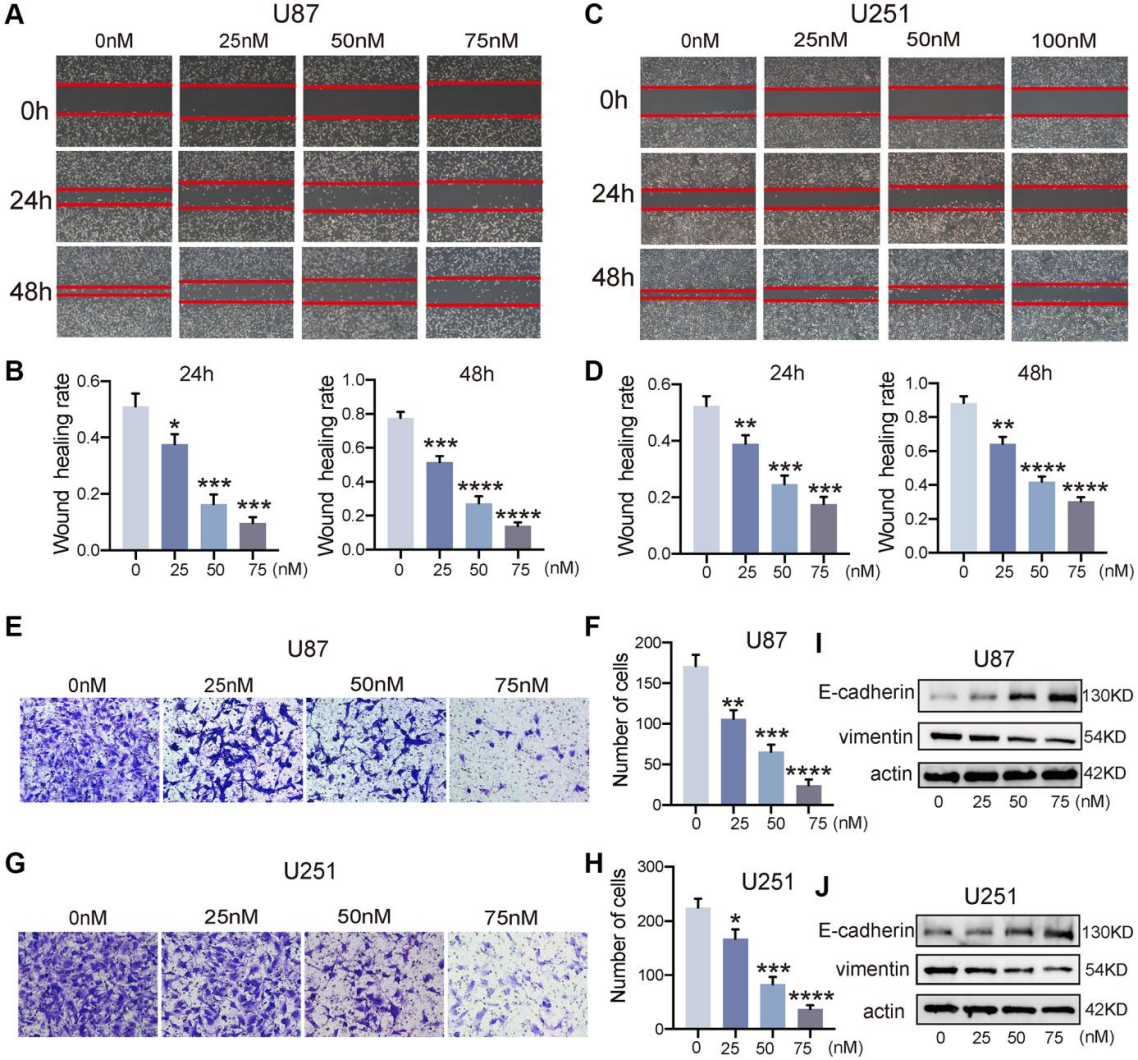
**Bufotalin attenuated the EMT process of GBM cells.** (**A**–**D**) The effect of bufotalin on U87 and U251 migration was assessed by wound healing assay at 24 h and 48 h. (**E**–**H**) The effect of bufotalin on U87 and U251 invasion was assessed by Transwell assay. (**I**, **J**) The expression levels of E-cadherin and Vimentin were analyzed by western blot. The data are representatives of three independent experiments and are presented as the mean ± SD. Significant differences compared with the control are indicated by ^*^p < 0.05, ^**^p < 0.01, ^***^p < 0.001, and ^****^p < 0.0001.

Building upon the aforementioned outcomes, we proceeded to explore the impact of bufotalin on the invasive and migratory capacities of GBM. First, we used wound-healing assay to examine the effect of bufotalin on the migration of GBM cells. The findings indicated that the migration of U87 and U251 cells was significantly inhibited by Bufotalin in a dose-dependent manner. Besides, the inhibitory effect of bufotalin on GBM cells was significantly stronger at 48 hours than at 24 hours (Figure 3A–3D). Consistently, the results of Transwell assays showed that bufotalin dose-dependently decreased the invasion of GBM cells (Figure 3E–3H). EMT has emerged as a crucial regulator of invasion, displaying a strong correlation with the malignancy of GBM [18]. To further investigate the effect of bufotalin on EMT, we performed western blotting to detect the expression of EMT-related genes, including E-cadherin and vimentin in GBM. The findings exhibited a dose-dependent escalation in E-cadherin expression subsequent to the administration of Bufotalin, coupled with a concomitant reduction in vimentin expression (Figure 3I, 3J). These findings suggested that Bufotalin attenuated the EMT in both U87 and U251 cell lines.

### Bufotalin induced GBM cells apoptosis *in vitro*

**Figure 4:**
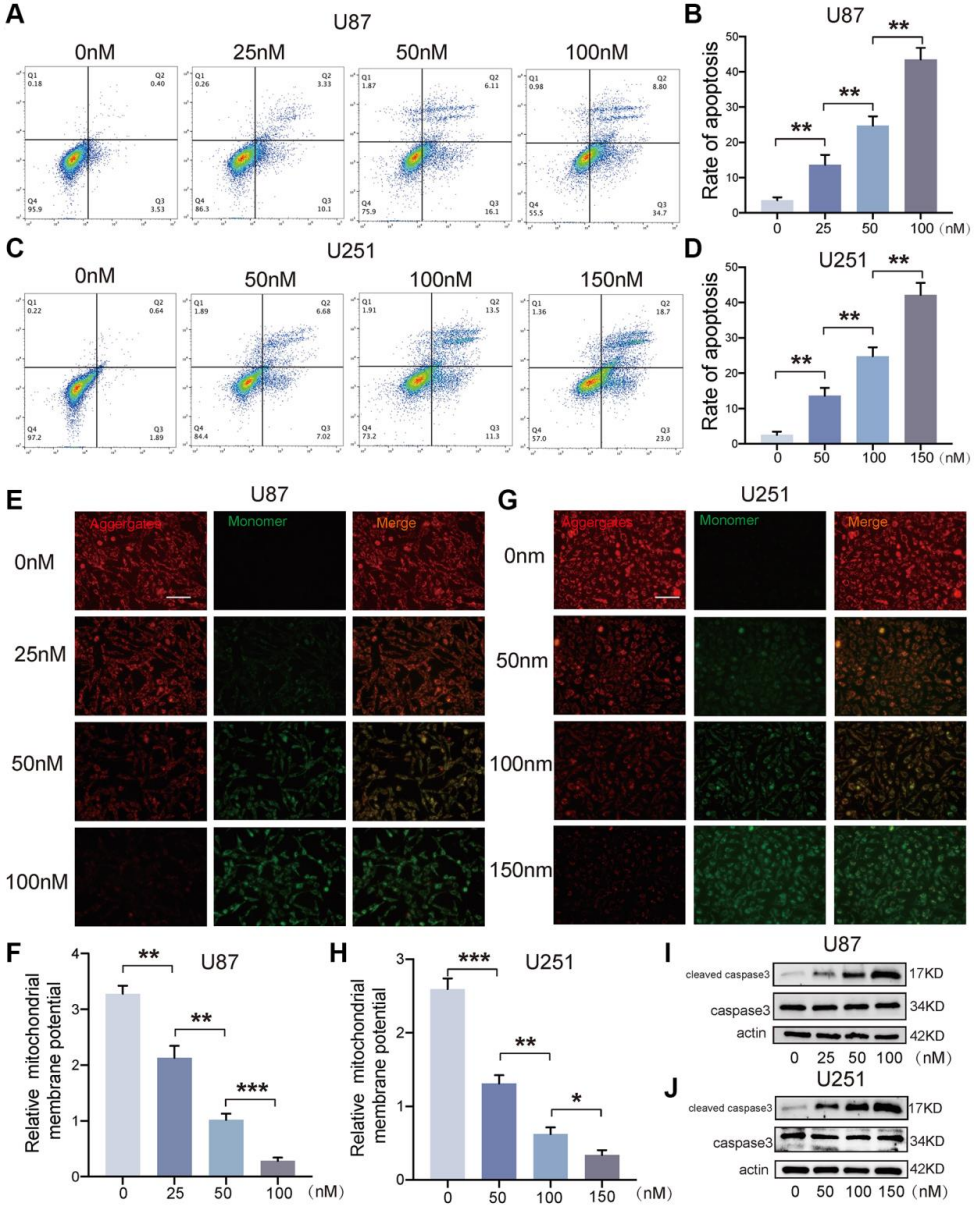
**Bufotalin induced GBM cells apoptosis *in vitro*.** (**A**–**D**) Cell apoptosis was measured by flow cytometry. U87 and U251 cells were treated with bufotalin for 24 h. (**E**–**H**) The changes in ΔΨm were monitored by JC-1 staining. (**I**, **J**) The expression levels of cleaved caspase-3 and caspase-3 were analyzed by western blot. Data are the mean ± SD of triplicate samples. The bar of the fluorescence image is shown in the figure. Significant differences compared with the control are indicated by ^*^p < 0.05, ^**^p < 0.01, and ^***^p < 0.001.

To detect the apoptosis of GBM cells, the cells which were treated with bufotalin after 24 h were stained with PE and 7AAD. Due to the different IC50 values of bufotalin on U87 and U251 cells, we treated these two cell lines with different concentrations of bufotalin. And the data showed that the rate of cell apoptosis significantly increased with the increase of bufotalin concentration (Figure 4A–4D). Besides, mitochondrial outer membrane permeabilization is an important step in the process of mitochondrial apoptosis induction, leading to the ultimate death of the cell [19]. Thus, we also performed JC-1 assays. Consistently, the results showed that bufotalin treatment decreased the ratio of red/green fluorescence intensity in both U87 and U251 cells (Figure 4E–4H). Caspase-3 is a commonly activated apoptotic protease, facilitating the selective cleavage of numerous pivotal cellular proteins, and mitochondrial dysfunction can activate caspase to start the process of cell death [20].

Thus, we detected the expression of cleaved caspase-3 and caspase-3 and found that bufotalin could promote the activation of caspase-3 in a dose-dependent manner in both U87 and U251 cells (Figure 4I, 4J). All these data demonstrated that bufotalin induced GBM cells apoptosis *in vitro*.

### Bufotalin promoted mitochondrial dysfunction by increasing ROS and decreasing the phosphorylation of AKT

**Figure 5:**
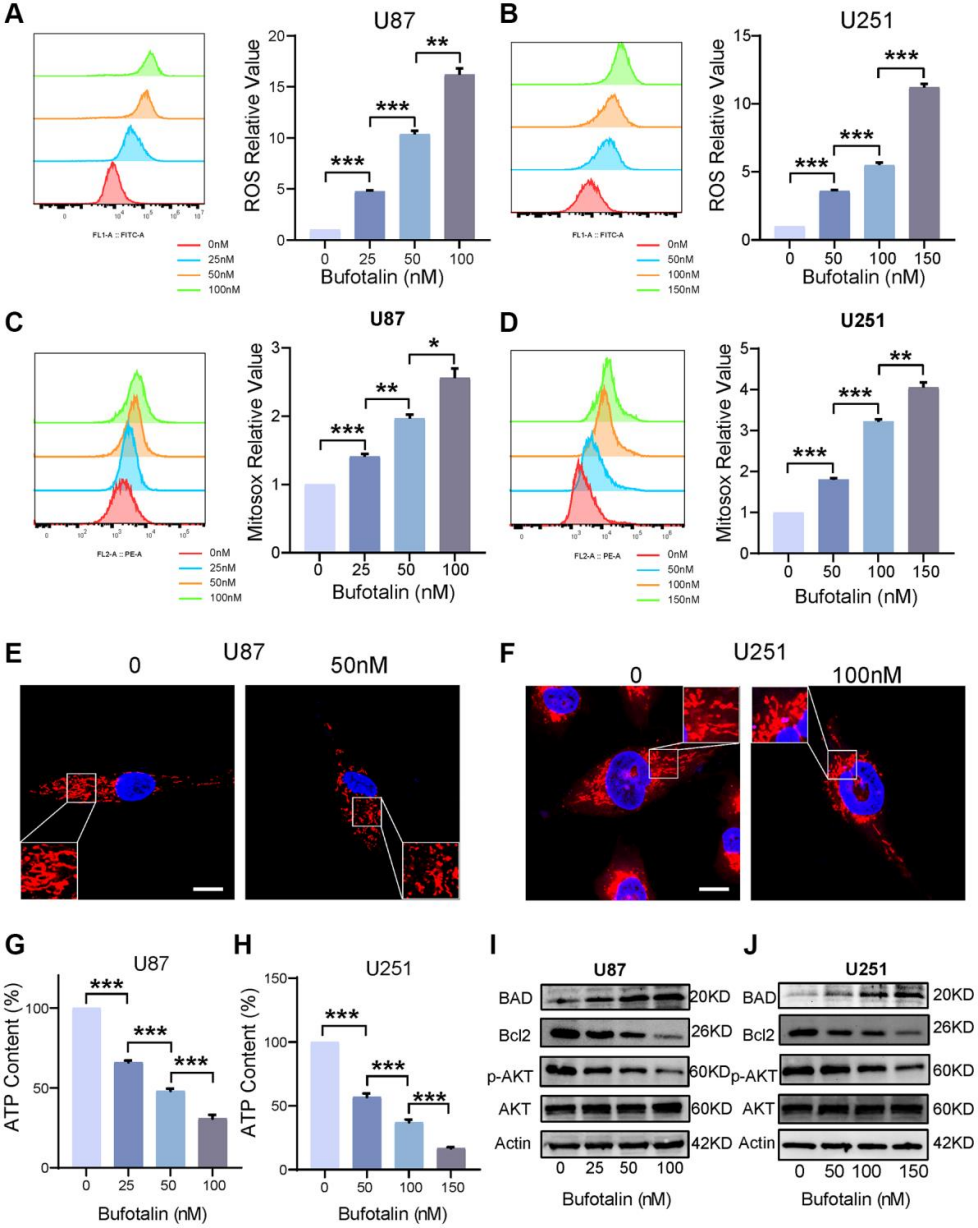
**Bufotalin promoted mitochondrial dysfunction by increasing ROS and decreasing the phosphorylation of AKT.** (**A**, **B**) ROS quantification was determined by flow cytometry using the H2DCFDA. (**C**, **D**) mitoROS quantification was determined by flow cytometry using the MitoSox. (**E**, **F**) Mitochondrial morphology was determined by Mito tracker using the confocal. (**G**, **H**) Decrease in ATP levels with increasing bufotalin concentration. (**I**, **J**) The expression levels of Bcl2, BAD, AKT and p-AKT were analyzed by western blot. Data are the mean ± SD of triplicate samples. Significant differences compared with the control are indicated by ^*^p < 0.05, ^**^p < 0.01, and ^***^p < 0.001.

We used flow cytometry to detect total ROS and found that the accumulation of intracellular ROS significantly increased with increasing bufotalin concentration (Figure 5A, 5B). Excessive accumulation of ROS can cause mitochondrial dysfunction [21]. Consistently, our study found that mitochondrial ROS significantly increased with a dose-dependent manner of bufotalin (Figure 5C, 5D). We also found that the mitochondria of the bufotalin-treated group had significant abnormalities compared to the control group by using confocal microscopy to observe the morphology of the mitochondria (Figure 5E, 5F). Furthermore, we detected a decrease in ATP levels with increasing bufotalin concentration (Figure 5G, 5H). All these results indicated that bufotalin disrupts mitochondrial function in GBM cells. Subsequently, we assessed the expression levels of Bcl2 and its associated proteins, pivotal in the regulation of mitochondrial function [22]. We found that bufotalin upregulated the expression level of BAD and downregulated the expression level of Bcl2. Besides, our data showed that the phosphorylation of AKT was downregulated by bufotalin (Figure 5I, 5J). All these results demonstrated that bufotalin promoted mitochondrial dysfunction by increasing ROS and decreasing the phosphorylation of AKT.

### Protecting mitochondria decreased the effect of bufotalin *in vitro*

**Figure 6:**
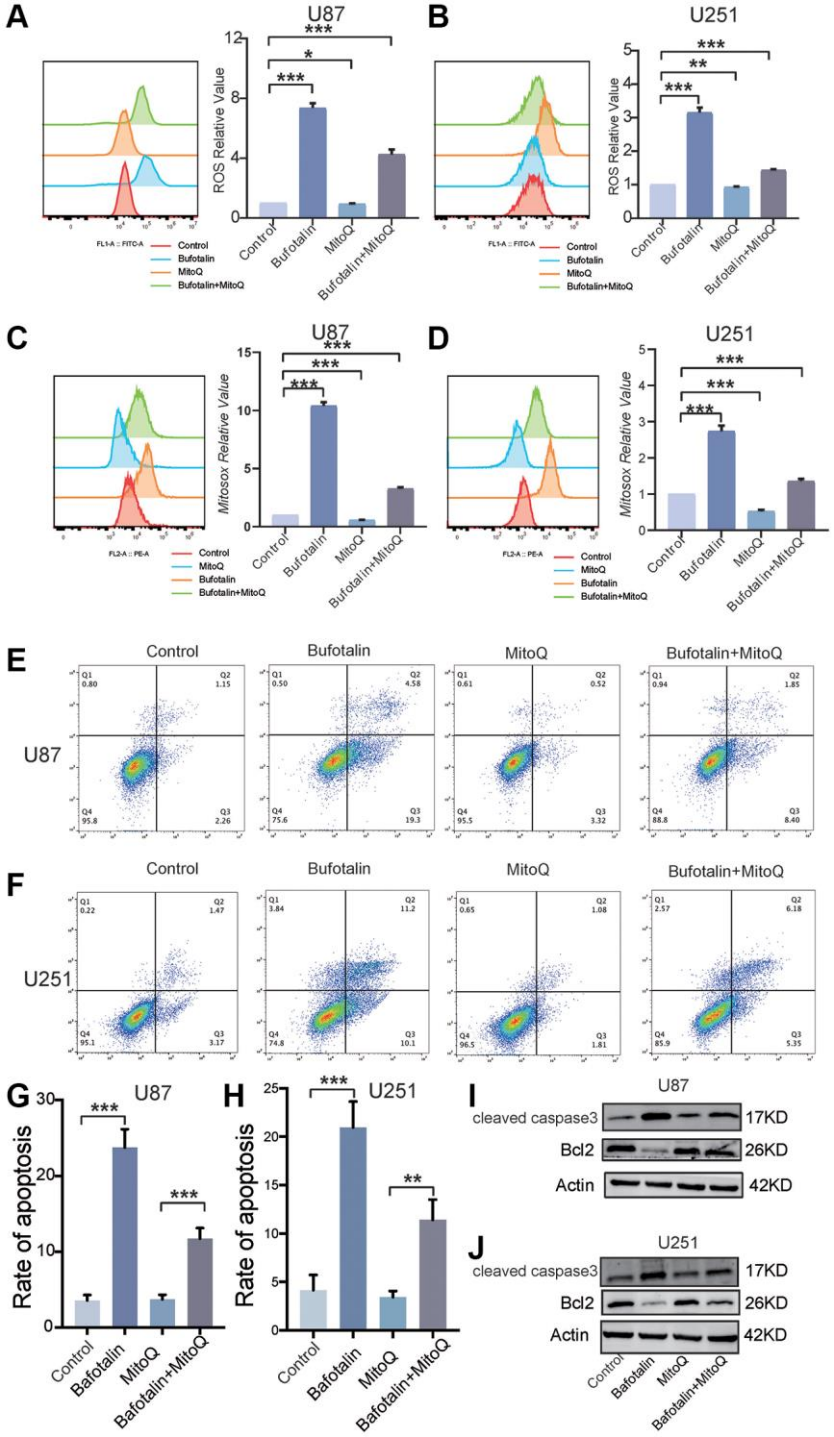
**Protecting mitochondria decreased the effect of bufotalin *in vitro*.** (**A**, **B**) ROS quantification of cells treated with MitoQ and bufotalin was determined by flow cytometry using the H2DCFDA. (**C**, **D**) mitoROS quantification of cells treated with MitoQ and bufotalin was determined by flow cytometry using the MitoSox. (**E**–**H**) Cell apoptosis was measured by flow cytometry. U87 and U251 cells were treated with MitoQ and bufotalin for 24 h. (**I**, **J**) The expression levels of Bcl2, cleaved caspase-3 and p-AKT were analyzed by western blot. Data are the mean ± SD of triplicate samples. Significant differences compared with the control are indicated by ^*^p < 0.05, ^**^p < 0.01, and ^***^p < 0.001.

Next, we used Mitoquinone (MitoQ) which is a mitochondria-targeted antioxidant [23] to rescue the effect of bufotalin in GBM cells. Furthermore, the results indicated a notable suppression by MitoQ of the increase in intracellular ROS induced by bufotalin (Figure 6A, 6B). Additionally, the elevation of mitochondrial ROS caused by bufotalin could be significantly inhibited by MitoQ (Figure 6C, 6D). Consistently, our study found that MitoQ could significantly reduce bufotalin-induced cell apoptosis (Figure 6E–6H). In addition, we observed that MitoQ reduces the changes in protein levels of caspase3 and Bcl2 induced by bufotalin (Figure 6I, 6J). These results indicated that bufotalin inhibits EMT and promotes apoptosis through inducing mitochondrial dysfunction in GBM cells.

### Bufotalin improved TMZ sensitivity of GBM cells *in vitro* and *in vivo*

**Figure 7:**
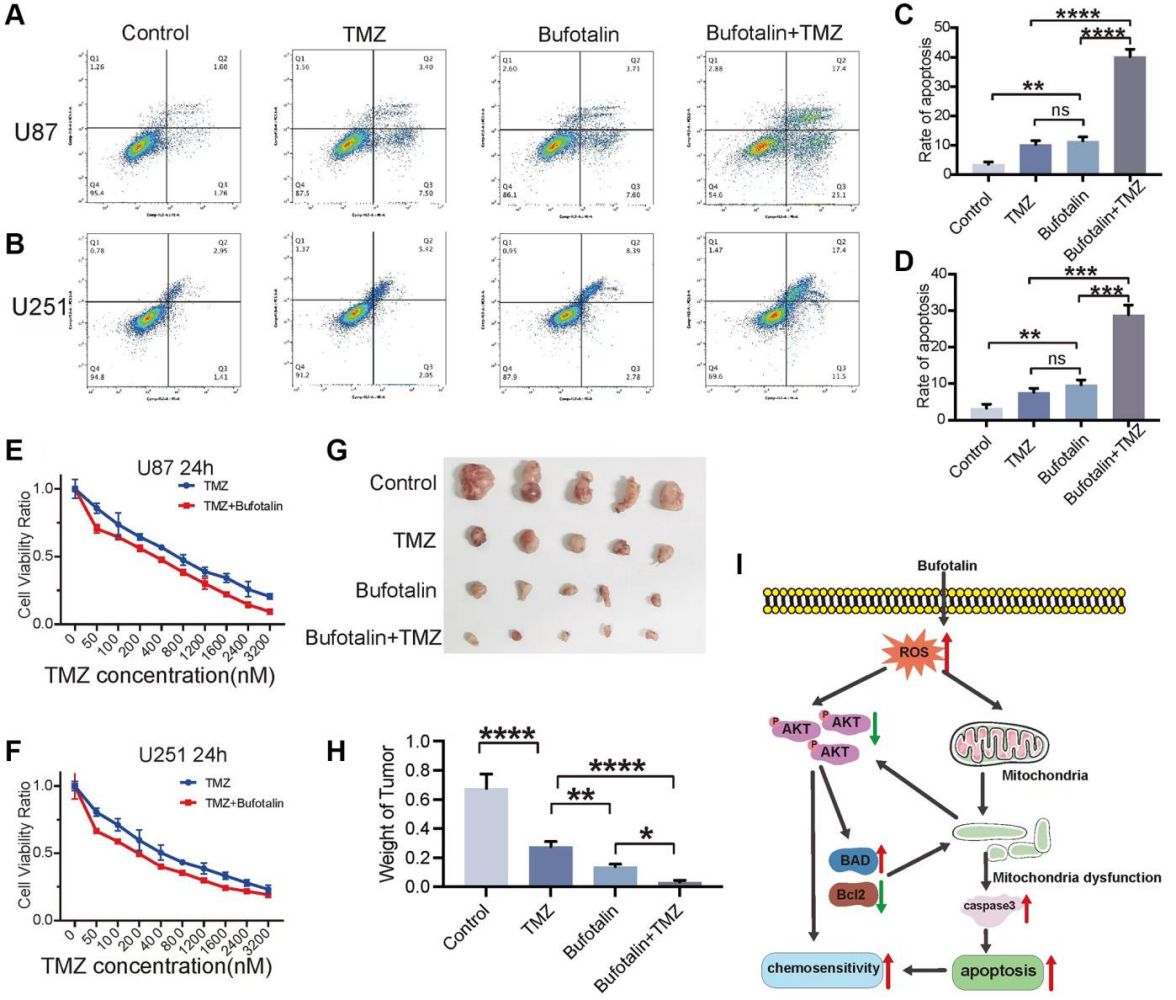
**Bufotalin improved TMZ sensitivity of GBM cells *in vitro* and *in vivo*.** (**A**–**D**) Cell apoptosis was measured by flow cytometry. U87 and U251 cells were treated with TMZ and bufotalin for 24 h. (**E**, **F**) CCK-8 assay was used to determine the cell viability of U87 and U251 cells treated with TMZ and bufotalin for 24 h. (**G**, **H**) Representative images and quantification of mouse tumors. (**I**) Schematic illustration of the molecular mechanism. Significant differences compared with the control are indicated by ^*^p < 0.05, ^**^p < 0.01, and ^***^p < 0.001.

Both EMT and apoptosis play a crucial role in the sensitivity of GBM to TMZ chemotherapy. First, we performed flow cytometry and the results indicated that the apoptosis rate of GBM cells induced by the combination of TMZ and bufotalin was significantly higher than the apoptosis rate when either drug was used alone (Figure 7A–7D). Consistently, our results of CCK8 also revealed that the IC50 of TMZ when used in combination with bufotalin was significantly lower than the IC50 of TMZ alone (Figure 7E, 7F). Moreover, the combination treatment of TMZ and bufotalin resulted in a notably reduced weight of the isolated tumors compared to the groups subjected to individual treatments with TMZ or bufotalin (Figure 7G, 7H). Therefore, our results demonstrate that bufotalin can enhance the chemosensitivity of GBM to TMZ.

## DISCUSSION

Bufotalin was identified as a type of bufadienolide, which showed potential anti-tumor effect in liver cancer, and melanoma. In this context, we first demonstrated the inhibitory effects of bufotalin on the proliferation and substantial attenuation of both proliferation and EMT in GBM cells, concurrently exhibiting a marked promotion of apoptosis. Furthermore, we also found that bufotalin could induce mitochondrial dysfunction by increasing the accumulation of ROS and inhibiting AKT phosphorylation. We further found that bufotalin can significantly enhance the chemosensitivity of GBM cells to TMZ (Figure 7I). Bufotalin is derived from the desiccated exudates of the auditory and cutaneous glands of Bufo gargarizans Cantor, which is used in the production of the traditional Chinese medicine ChanSu [24]. In recent years, more and more studies have revealed its anti-tumor effects in various types of cancer. Su et al. observed that bufotalin triggered apoptotic processes in human hepatocellular carcinoma Hep 3B cells. However, the effect could be obviously reduced by caspase inhibitor, which indicated that caspases were involved in bufotalin-induced apoptosis of Hep 3B cells [25]. In addition, bufotalin administration resulted in cell cycle arrest at the G2/M phase, which was achieved by reducing the expression levels of CDK1, CDC25, cyclin A and cyclin B1, while simultaneously increasing the levels of p53 and p21 [26], it also has similar effects in melanoma cells [16]. Moreover, bufotalin caused the activation of endoplasmic reticulum (ER) stress in osteoblastoma cells through the induction of CHOP and phosphorylation of IRE1 [27]. Bufotalin enhances the apoptosis of HeLa cells induced by TTRAIL and TNF-a. It achieves this by sensitizing death receptor-mediated apoptosis through Bid- and STAT1-dependent pathways [28]. In our study, we first ensured that bufotalin could inhibit EMT and inhibit apoptosis of GBM cells. According to the results of RNA-seq, we found that these effects were achieved through the dysfunction of mitochondria via the treatment of bufotalin.

ROS regulate various aspects of biological processes, and their abnormal production and metabolism are crucial characteristics of GBM cells. For instance, ROS can enhance the transcription of vascular endothelial growth factor (VEGF) by stabilizing HIF-1α, thereby inducing angiogenesis and promoting the growth of GBM [29]. Besides, ROS could also induce MMP-9 expression through activation of extracellular signals, thereby enhancing invasion and migration of U87 glioma cells [30]. However, ROS is a double-edged sword in GBM cells. When it accumulates excessively, the antioxidant system is unable to neutralize the excess ROS, leading to severe damage to cell organelles and DNA, thereby causing cellular damage and inducing cell death [31]. Thus, modulating ROS may be an effective strategy for the treatment of GBM. In our study, it showed that bufotalin could significantly promote the levels of ROS in GBM cells. As is widely known, the excessive accumulation of ROS in tumor cells will induce anti-tumor effects by initiating programmes associated with regulated cell death (RCD), which mainly includes apoptosis, ferroptosis and necrosis [32]. For example, ROS accelerate ubiquitin-mediated proteasomal degradation of the anti-apoptotic factor FLICE inhibitory protein, thereby stimulating the extrinsic apoptotic pathway, which inhibits DISC formation by competitively binding to adaptor proteins and procaspase-8 [33]. Furthermore, the excessive accumulation of ROS can modulate the permeability of the outer mitochondrial membrane through the regulation of the Bcl-2 family. This modulation leads to the liberation of Cyt-c, which subsequently engages with apoptosis protease activating factor 1 and caspase-9, culminating in the formation of an apoptosome. The apoptosome, in turn, triggers the caspase-9 signaling cascade, instigating the process of apoptosis [34]. In our study, we found that bufotalin could induce mitochondrial dysfunction via overproduction of ROS which could change mitochondrial membrane permeability and regulate protein such as Bcl2 or BAD. Besides, we also found that bufotalin could also decrease the expression of Bcl2 family proteins by inhibiting the phosphorylation of AKT, which further enhances mitochondrial dysfunction.

Mitochondrial dysfunction is a characteristic of cancer [35]. In glioblastoma, alterations in mitochondrial genes result in substantial impairment of mitochondrial function, giving rise to both morphological and bioenergetic abnormalities. These abnormalities encompass heightened production of ROS. Besides, elevated mitochondrial reactive oxygen species (mtROS) from dysfunctional mitochondria trigger oxidative stress by activating downstream pathways of p53 and the Bcl-2 family proteins, ultimately leading to cell apoptosis [11, 36]. In our study, we found that bufotalin could significantly increase total ROS and also cause abnormal elevation of mtROS, leading to mitochondrial dysfunction, such as reduced ATP synthesis and morphological changes, thereby inhibiting EMT and promoting apoptosis of GBM cells. Moreover, we also found that the excessive accumulation of ROS induced by bufotalin can inhibit the phosphorylation of AKT, which plays an important role in inhibiting apoptosis in GBM cells. In glioma, the activation of AKT governs various biological processes associated with cellular survival and growth. The engagement of the PI3K/AKT pathway has been associated with the degree of malignancy in gliomas [37]. Therefore, inhibiting the activity of AKT is of significant importance for the treatment of GBM. For instance, Sempervirine induces G2/M phase cell cycle arrest and promotes cell apoptosis by inhibiting the AKT/mTOR signaling pathway [38]. Besides, inhibiting the AKT signaling pathway can enhance the sensitivity of GBM to TMZ [39, 40]. Inhibition of mitoSTAT3 activation and induction of mitochondrial respiratory dysfunction could increase the sensitivity of GBM cells to TMZ [41]. Excessive production of ROS could not only cause mitochondrial dysfunction by interfering with the expression of the Bcl-2 family, but also enhances DNA damage to further increase the chemosensitivity of GBM cells to TMZ [42]. In our study, we found that bufotalin could enhance the anti-tumor effects of TMZ in GBM cells, which may be induced by downregulating phosphorylation of AKT and mitochondrial dysfunction via ROS.

In summary, we have a preliminary conclusion on the potential mechanism of bufotalin as a treatment for GBM: by inducing excessive accumulation of intracellular ROS and mitochondrial dysfunction, it affects the AKT signaling pathway, thereby inhibiting the proliferation and EMT process of GBM cells. Specifically, bufotalin inhibits AKT phosphorylation by affecting the increase of ROS content in GBM cells, thereby regulating the expression of Bcl2 family proteins, leading to the activation of caspase-3 and inducing cell apoptosis. Meanwhile, bufotalin can directly cause mitochondrial damage, promote the activation of caspase-3, and induce cell apoptosis.

## CONCLUSION

In summary, we demonstrated that bufotalin, a type of bufadienolide, was capable of substantially inhibiting EMT as well as promoting apoptosis in GBM cells by inducing mitochondrial dysfunction and decreasing the phosphorylation of AKT via accumulation of ROS. Besides, bufotalin could significantly enhance the chemosensitivity of GBM cells to TMZ *in vitro* and *in vivo*. These results showed that bufotalin has strong anti-tumor abilities and can provide significant value for the treatment of GBM patients.

## MATERIALS AND METHODS

### Cell culture

The U87 and U251 cell lines were obtained from the Cell Bank of the Shanghai Institute of Biochemistry and Cell Biology located in Shanghai, China. These cells were grown in high glucose DMEM and supplemented with 10% fetal bovine serum and 1% penicillin/streptomycin. The cells were incubated at 37°C with 5% CO_2_.

### Reagents and antibodies

The reagents and antibodies that were used in our study included: bufotalin (HY-N0878, MCE, USA); Actin (AC026, Abclonal, China), Bcl2 (381702, Zenbio, China), BAX (R380709, Zenbio, China), BAD(R23582, Zenbio, China), caspase-3(A2156, Abclonal, China), cleaved caspase-3 (A19654, Abclonal, China), AKT (382804, Zenbio, China), p-AKT(310021, Zenbio, China), HRP Goat Anti-Rabbit IgG (AS014, Abclonal, China), HRP Goat Anti-Mouse IgG (AS003, Abclonal, China) and Cy3 Goat Anti-Rabbit IgG(AS007, Abclonal, China).

### Apoptosis analysis

The apoptosis rate of GBM cells was assessed with Annexin V-PE/7-AAD kit (Becton Dickinson, USA). GBM cells were collected and washed with PBS for three times. Subsequently, the cells were stained with Annexin V-PE/7-AAD for 15 minutes in the absence of light. Then the specific apoptosis of GBM cells was analyzed by CytoFlex (Beckman, USA). Cells in the early stage of apoptosis exhibited positive staining for Annexin V-PE and negative staining for 7-AAD and cells in the late stage of apoptosis or already dead displayed positive staining for both 7-AAD and Annexin V-PE. To determine the total apoptosis rate and perform statistical analysis, the sum of the upper and lower right quadrants was calculated.

### Transwell assay

The Transwell assay was conducted using Transwell chambers and polycarbonate membranes coated with Matrigel (R&D, USA). The cells were treated with different concentrations of bufotalin and then seeded in the upper chamber with 200 μl of serum-free medium. In the lower chamber, 600 μl of medium with 10% FBS was added as a chemoattractant. The cells were incubated at 37°C and 5% CO_2_ for 24 hours in an incubator. After fixation with 4% paraformaldehyde, the cells on the lower side of the Transwell chamber were stained with 0.5% crystal violet. The image was captured by Olympus BX71 (Japan).

### Wound healing assay

After the cells were seeded in a 6-well plate, 200 μL sterile pipette tips were used to scrape the cells. Then the cells were cultured with a medium containing different concentrations of bufotalin and 1% FBS to reduce the effect of proliferation. At 0 h, 24 h, and 48 h, the image of the wound was captured by Olympus BX71. And ImageJ was used to quantify the area of the wound, enabling the calculation and analysis of the wound-healing percentage.

### Colony assay

To conduct colony-formation assays, 600 cells were distributed into six-well plates and then were incubated at 37°C and 5% CO_2_. After two weeks, 4% paraformaldehyde was used to fix the GBM cells for 15 min and they were stained with 0.1% crystal violet for 30 min. The representative colonies were photographed and quantified.

### CCK-8 assay

Cells were seeded in a 96-well plate (6000 cells per well) and treated with different concentrations of bufotalin and TMZ. After 24 or 48 h, the culture media was removed from the wells and the CCK-8 reagent (Beyotime, China) was added to each well. The plate was incubated for an hour at 37°C in an incubator. After the incubation, we measured the absorbance of each well at 450 nm using Multimode Plate Reader (PerkinElmer, Germany), analyzed the obtained absorbance values to determine cell viability or proliferation rates, and calculated the percentage of cell viability or growth inhibition compared to the control group.

### ATP assay

After the cells were treated with different concentrations of bufotalin, 200 μL of lysis buffer was added to each well of a 6-well plate to lyse the cells. 100 μL of ATP detection working solution (Beyotime, China) was added to each well of a 96-well plate and then 20 μL sample was added and mixed well quickly [43]. Multimode Plate Reader (PerkinElmer, Germany) was used to measure RLU of each well.

### Total ROS detection and MitoSox assay

To detect the Total ROS, cells were labeled with the DCFDA Cellular ROS detection assay kit (Beyotime, China) following the manufacturer’s protocol. In brief, cells which were treated with different concentrations of bufotalin were stained with 20 μM DCFDA for 60 min in the incubator. For MitoSox assay, cells were labeled with MitoSox Red mitochondrial superoxide indicator (Invitrogen, USA) in 5 μM concentration for 30 min at 37°C in the dark. After incubation, cells were trypsinized and washed with PBS two times and analyzed by CytoFlex (Beckman, USA).

### RNA-seq

U87 cells were treated in triplicates with 100 nM bufotalin in DMEM medium for 24 h. DMEM medium was used as the control treatment. After the treatment, total RNA was extracted using TRIzol. For RNA-seq, library construction and sequencing were performed by Biomarker Technologies (Beijing, China). The Volcano Plot, heat map of differentially expressed genes (DEGs) and GSRA were performed by R. Genes with an adjusted *p*-value of < 0.05 were considered to be differentially expressed.

### Mitochondrial membrane potential (ΔΨm) assay

The decrease in mitochondrial membrane potential is a hallmark event in the early stages of cell apoptosis [44]. The transition from red fluorescence to green fluorescence with JC-1 can easily detect the decrease in cell membrane potential, and it can also be used as an indicator for early detection of cell apoptosis. The concentration of JC-1 working solution was 5 ng/ml and was added into each well after the cells were treated with different concentrations of bufotalin. Then cells were incubated for 20 min at 37°C. The images were captured by Olympus BX71 immediately after the staining.

### MitoTracker red staining

Mito-Tracker specifically labels mitochondria with biological activity in cells and detects mitochondrial membrane potential. After the cells were treated with different concentrations of bufotalin, MitoTracker Red (Beyotime, China) was added into each well after removing the medium and then the cells were incubated with working solution for 30 min at 37°C [45]. In addition, DAPI was used to stain the nuclei. The images were captured by confocal microscope immediately after the staining.

### Western blot

Total protein was obtained from cells which were lysed using RIPA lysis buffer. Then the proteins were separated via SDS-PAGE and transferred onto a PVDF membrane (Millipore, Germany). After blocking with 5% skim milk, the PVDF membranes were incubated in the primary antibody at 4°C overnight. Following this, the membranes were incubated with corresponding secondary antibodies for 1 hour, and the band densities were detected using the ECL Chemiluminescence Kit. The image was captured using a ChemiDoc Touch (Bio-Rad, USA).

### Construction and tumor measurement of Xenograft model

U87 cells were injected subcutaneously into the axilla of a 4-week-old nude mice (*n* = 5/group). Two weeks after cell planting, the mice were treated with TMZ, Bufotalin and TMZ combined with bufotalin respectively by intraperitoneal injection every 2 days for 5 times. Mice were euthanized at 28 days after cell injection to obtain tumor weights. All procedures and experiments on animals were approved by the Institutional Animal Care and Use Committee subordinate to Renmin Hospital of Wuhan University.

### Statistical analysis

The mean ± SD of three independent experiments was used to present all data. Student’s *t*-test was employed to assess the statistical significance for two groups, while one-way ANOVA was utilized for multiple groups. Prism 8 software (GraphPad Software Inc., USA) was used for these analyses. Significance levels were determined as ^*^p < 0.05, ^**^p < 0.01, and ^***^p < 0.001.

### Data availability

Data will be made available on request.
